# Hot Carrier Nanowire Transistors at the Ballistic Limit

**DOI:** 10.1021/acs.nanolett.4c01197

**Published:** 2024-06-24

**Authors:** Mukesh Kumar, Ali Nowzari, Axel R. Persson, Sören Jeppesen, Andreas Wacker, Gerald Bastard, Reine L. Wallenberg, Federico Capasso, Ville F. Maisi, Lars Samuelson

**Affiliations:** † NanoLund and Division of Solid State Physics, Lund University, Box 118, 22100 Lund, Sweden; ‡ NanoLund and Centre for Analysis and Synthesis, Lund University, Box 117, 22100, Lund, Sweden; ¶ NanoLund and Division of Mathematical Physics, Lund University, Box 118, 22100 Lund, Sweden; § Physics Department ENS-PSL, Laboratoire Pierre Aigrain LPA, 24 Rue Lhomond F75005 Paris, France; ∥ John A. Paulson School of Engineering and Applied Sciences, Harvard University, 9 Oxford Street McKay Laboratories, Room 125, Cambridge, Massachusetts 02138, United States; ⊥ Institute of Nanoscience and Applications, Southern University of Science and Technology, 1088 Xueyuan Avenue, Shenzhen 518055, China

**Keywords:** hot carrier transistors, ballistic electrons, bandgap engineering, quantum mechanical transmission

## Abstract

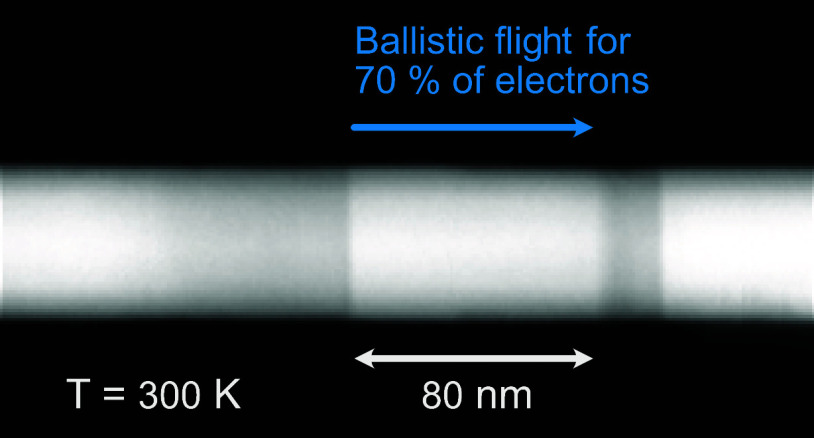

We demonstrate experimentally nonequilibrium transport in unipolar quasi-1D hot electron devices reaching the ballistic limit at room temperature. The devices are realized with heterostructure engineering in nanowires to obtain dopant- and dislocation-free 1D-epitaxy and flexible bandgap engineering. We show experimentally the control of hot electron injection with a graded conduction band profile and the subsequent filtering of hot and relaxed electrons with rectangular energy barriers. The number of electrons passing the barrier depends exponentially on the transport length with a mean-free path of 200–260 nm, and the electrons reach the ballistic transport regime for the shortest devices with 70% of the electrons flying freely through the base electrode and the barrier reflections limiting the transport to the collector.

Hot carriers open up many interesting device concept paradigms based on their ballistic transport in semiconductors.^1,2^ In the ballistic transport, a significant fraction of the charge carriers move through the structure without losing their energy. Thus, the highest speed operation is potentially feasible since many types of scattering have not yet limited the carrier flow. To obtain hot electron devices, the bandgap energy of a semiconductor needs modulations at energies much larger than the thermal energy *kT*, to generate and detect highly nonequilibrium electrons. Typically, bandgap engineering for generating hot electrons has been done by either gating,^3−5^ doping,^6,7^ or using Schottky or tunnel barriers in metal–oxide–semiconductor structures.^8−13^ More recently, monolayer materials have also been employed to obtain extremely short base electrodes^14−16^ and III-nitrides with large bandgap.^17−20^ Bandgap engineering is also used extensively in modern electronics. For example in any cell phone receiver/transmitter block, 2D epitaxy of graded SiGe thin base layers is controlled at the atomic level by chemical vapor deposition (CVD), yielding the best performance.^21,22^ While successful in making ballistic devices, the above-mentioned approaches come with major constraints. For doped structures, the dopants act as scattering centers. Additional scattering and energy relaxation therefore limit the free flight of the carriers, which is a serious constraint for the ballisticity. Doping also typically varies smoothly in the structure, preventing sharp boundaries between different operational parts of the devices. On the other hand, the Schottky barrier-based devices generate hot electrons via a tunneling process. This together with the inability to adjust the barrier height and thickness hinders choosing and setting the hot carriers and their detector barrier to specific energies. In this paper, we use 1D epitaxy in semiconductor nanowires for bandgap engineering. The 1D epitaxy allows us to tailor the bandgap of a III–V semiconductor by combining different lattice-mismatched materials into heterostructures free from defects and dopants thanks to the efficient radial relaxation in the nanowire geometry.^23−28^ 1D epitaxy is also beneficial for reducing the possible scattering directions compared to the planar 2D/3D geometries. We make devices with 1D epitaxy where up to 70% of the electrons fly ballistically at room temperature and determine the mean-free part and the reflection probability for the electrons at the barrier. The findings are consistent with the theoretical predictions.

The conduction band profile of our hot electron injector–collector system is presented in Figure 1a. A graded potential barrier on the left forms a hot electron injector. Electrons will be injected close to the maximum value of the barrier by raising the chemical potential of the graded side; see Figure 2b, which flattens the graded barrier and allows electrons to flow through this graded segment. As a detector, we use a rectangular barrier that has the same height as the injector so that ideally only ballistic electrons at a high energy pass. Electrons that relax in the middle region will be blocked by the rectangular barrier, which we grow thick enough (thickness 20 nm with a barrier height of approximately 500 meV in the InAs/InP heterointerface) to suppress tunneling at low energies. Thus, the electrical current after the filtering barrier measures the number of ballistic electrons that fly over the barrier. The sharp interfaces also define the length *l* of the base electrode between the injector and filter unambiguously.

**Figure 1:**
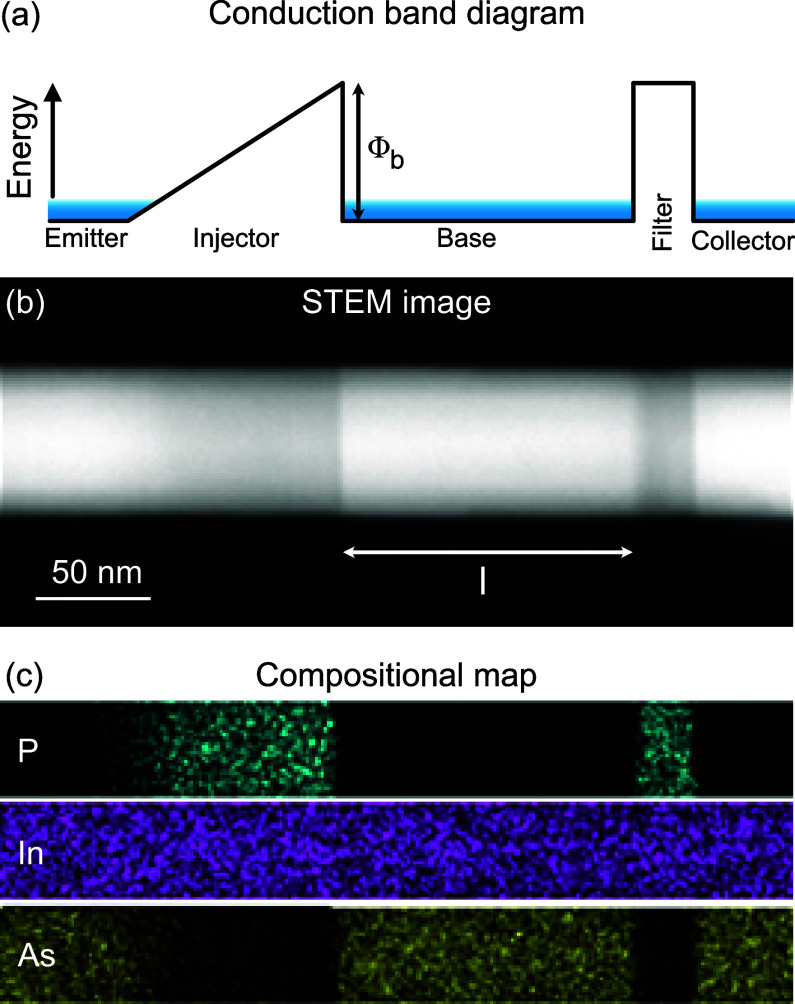
(a) The conduction band diagram of the ballistic hot electron system consisting of a graded potential barrier as a hot electron injector and a rectangular barrier for filtering the electrons. (b) Scanning transmission electron microscope image of a nanowire heterostructure obtained through growth engineering showing an InAs_1–*x*_P_*x*_-based quasi-1D hot electron injector and InP-based energy filter. (c) Compositional map of In, As, and P along the nanowire.

**Figure 2:**
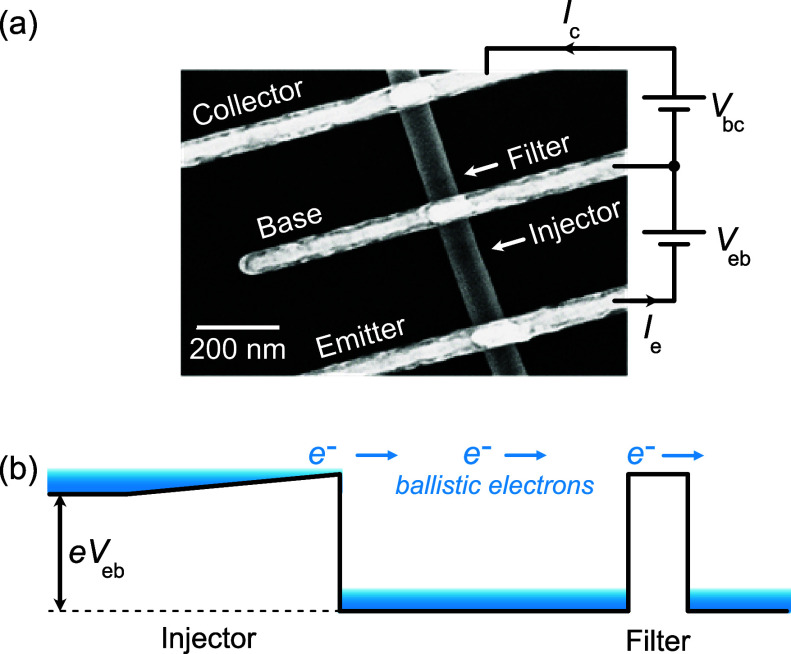
(a) A scanning electron micrograph showing one of the measured unipolar 3-terminal hot electron nanowire devices. The middle base electrode needs to be positioned and defined sharply between the injector and the energy filter, spaced by a distance *l*. Voltage *V*_eb_ biases the injector and *V*_bc_ the filter. (b) Band diagram under typical operation. The voltage *V*_eb_ lifts the energy of the emitter electrons and flattens the graded barrier, giving rise to electron injection. Ballistic electrons fly over the filter to an unbiased (*V*_bc_ = 0) collector.

For realizing the band diagram of Figure 1a, we grow InAs/InAsP nanowire heterostructures with chemical beam epitaxy (CBE), allowing for the demanding abrupt heterointerfaces^29^ and smooth graded barriers^28^ within the same growth run. The InAs and InP have additionally a large conduction band offset, low effective mass, and high electron mobility, which are preferred properties for the hot electron devices.^30^ The graded injectors in InAs-based nanowires were formed with graded InAs_1–*x*_P_*x*_ through in situ modulation of chemical composition *x* along the length of nanowires, while the rectangular filter barrier was made with fixed *x*; see Supporting Information for further details. Figures 1b and 1c show a scanning transmission electron micrograph and compositional maps of the structure that highlight clearly the key features and dimensions. To estimate the barrier height for both the injector and filter, we estimate the uppermost phosphorus content to be around *x* ≈ 0.8 in both segments, corresponding to a conduction band offset of about 0.5 eV.^28,30^

After growth, we transferred the structures to a Si/SiO_2_ chip and made ohmic contacts. We made three contacts: an emitter contact before the injector, a collector contact after the rectangular barrier, and a common base contact in between. These form a transistor configuration, as presented in Figure 2. We used electron beam lithography to define and position the contacts, allowing the fabrication of devices with base lengths down to *l* = 80 nm. Before the deposition of the 25/125 nm Ni/Au contacts with thermal evaporation, sulfur passivation^31^ was used for obtaining low contact resistance on the order of 100 Ω measured from similar contacts made to the plain InAs segment.

The generation of ballistic electrons in the structure takes place by applying a bias voltage, *V*_eb_, to the graded barrier. This voltage lifts the electrons on the left of the injector to higher energies and flattens the graded barrier as shown in Figure 2b. When the energy from the bias voltage *eV*_eb_ exceeds the barrier height Φ_b_, the injector barrier no longer limits the current flow, leading to high injection current with electrons at energy Φ_b_ in the base region. We measure the injection current *I*_e_ from the emitter side. The energetic electrons have two possible scenarios at the base. Either they continue at high energy over the filtering barrier contributing to collector current *I*_c_, or they relax at the base regime, get trapped there, and flow away to ground from the base contact.

Figure 3 presents transport data for the device of Figure 2 with *l* = 150 nm. We indeed see vanishing injector current *I*_e_ at low bias voltage, and at *V*_eb_ > 0.5 V, the current increases steeply, consistent with the estimated barrier height of Φ_b_ ≈ 500 meV. These findings and numbers are consistent with our earlier study of the graded barrier as electrical diodes.^28^ On the collector side, the current *I*_c_ stays also vanishingly small at low *V*_eb_ and increases proportionally to the emitter current *I*_e_. The inset presents the proportionality as the transfer ratio *I*_c_/*I*_e_. We observe that the proportionality stays within *I*_c_/*I*_e_ = 14 ± 2% over the whole injection range. Interestingly, if we apply a large reverse bias *V*_eb_ < −0.6 V to the emitter, a small leakage current *I*_e_ appears at the emitter, but no current is induced at the collector side. This reverse biasing removes equilibrium electrons at low energies from the base and does not create high-energy ones. As the collector current remains at *I*_c_ = 0, the collector side does not respond to these low-energy excitations. As a further proof of the energy selectivity, Figure 4 shows the collector current *I*_c_ as a function of the base collector voltage *V*_bc_ for different injection voltages *V*_eb_. For *V*_eb_ < 0.2 V, all the curves stack on top of each other with a diminishing collector current *I*_c_ below the 0.02 nA level up to *V*_bc_ = ±50 mV. Hence, we have no injection from the emitter here. At larger *V*_bc_, an exponentially increasing leakage current *I*_c_ through the energy filter arises, as seen in the yellow data with *V*_eb_ = −0.8 V in the top panel. The exponential dependence arises because the bias voltage *V*_bc_ slants toward the rectangular filter barrier profile, leading to a lower average barrier height. This increases the tunneling probability and hence the current exponentially for the low-energy electrons.^32,33^ By using *V*_bc_ = 0 for the injection experiments, we minimized these leakage contributions. With emission, the collector current *I*_c_ depends only weakly on the collector bias voltage *V*_bc_: a bias voltage variation of *V*_bc_ = ±50 mV changes the collector current by 20% or less in the high emission current regime.

**Figure 3:**
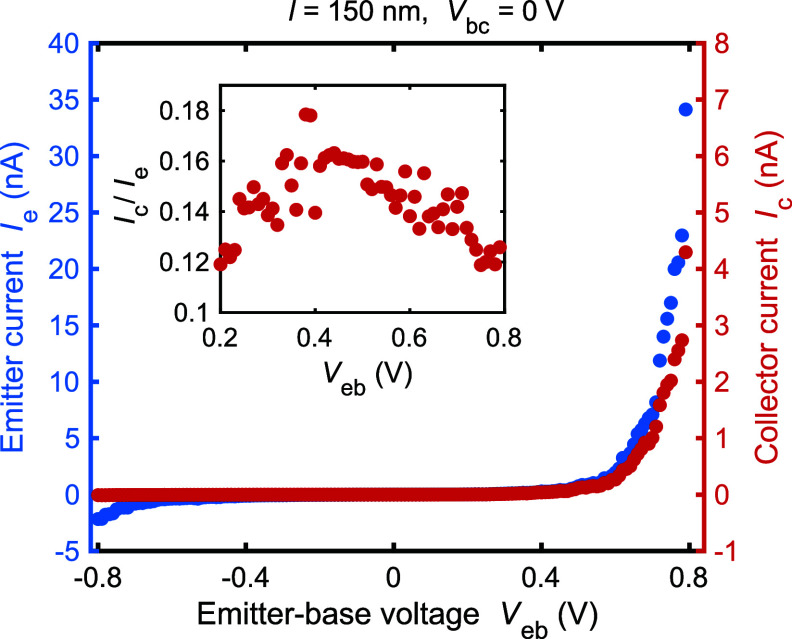
Measured current–voltage curves for a device with a base length *l* = 150 nm. The emitter current *I*_e_ and collector current *I*_c_ were measured simultaneously as a function of the injector voltage *V*_eb_. The energy filter was kept unbiased at *V*_bc_ = 0. The inset shows the transfer ratio *I*_c_/*I*_e_ at the injection regime. All measurements took place at room temperature.

**Figure 4:**
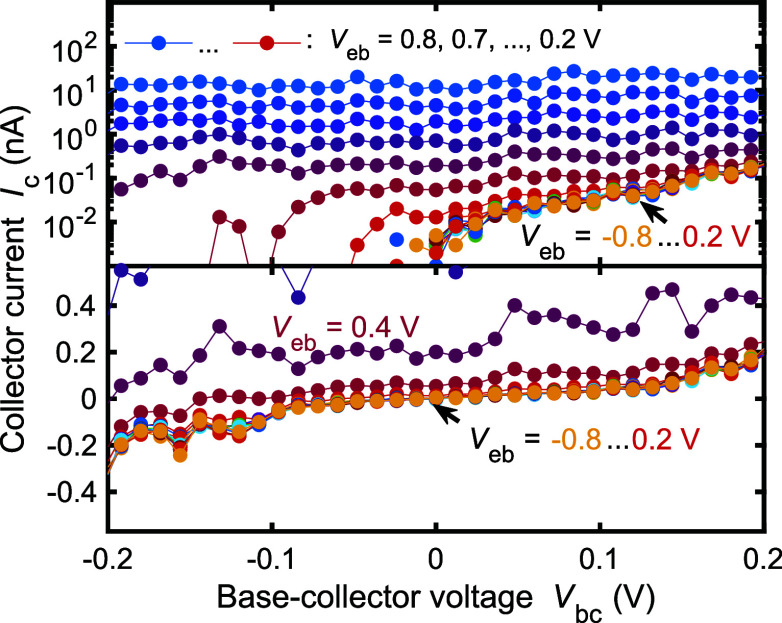
Collector current *I*_c_ as a function of the base-collector voltage *V*_bc_ for emitter-base voltages *V*_eb_ = −0.8, ..., 0.8 V. The top panel shows the current on a logarithmic scale, and the bottom panel repeats the data with a linear scale. The device has a base length of *l* = 160 nm.

We now turn to investigating the base length *l* dependence in order to study the ballistic transport characteristics through the base and over the rectangular barrier. For that, we repeated the device fabrication and measurements for varying base lengths. Figure 5 summarizes the findings. We observed consistently a collector current that was proportional to the injection current and depended only weakly on the other parameter values, as above. However, the transfer ratio *I*_c_/*I*_e_ depends strongly on the base length *l*. We see from Figure 5 that the measured *l* = 80–2000 nm range results in more than 4 orders of magnitude change to *I*_c_/*I*_e_ and scales exponentially as *I*_c_/*I*_e_ = *T* exp(−*l*/*l*_r_), as shown by the solid lines fitted to the data. From the fit, we determine energy relaxation length *l*_r_ = 220 nm and transmission probability *T* = 0.28 through the filter for ballistic electrons. For the shortest devices with *l* = 80 nm, we obtain exp(−*l*/*l*_r_) = exp(−80 nm/220 nm) = 70% of the electrons ballistically flying to the filter. Here the base length with near-unity ballistic transport is more than an order of magnitude larger than the typical values for room-temperature ballistic devices, such as monolayer-based structures^14−16^ and GaN transistors with a typical base length of 10 nm.^19,20^ Furthermore, the transmission probability *T* = 0.28 sets predominantly the transfer ratio *I*_c_/*I*_e_ = 0.19 in our devices. In other words, *R* = 1 – *T* = 72% of the ballistic electrons are reflected at the base–filter barrier interface. The observed transmission probability *T* and reflection probability *R* are consistent with theoretical values based on quantum mechanical reflection calculation: with a rectangular barrier (Φ_b_ = 500 meV, thickness *a* = 20 nm) a thermal electron distribution above the barrier provides an average transmission of *T* = 0.3, in good agreement with the measured value;^32,33^ see methods. Therefore, quantum mechanics explain the transmission probability *T* of our system and why the transfer ratio is limited to *I*_c_/*I*_e_ = 0.19, equaling the product of 70% ballistic propagation probability and *T* = 0.28 transmission probability through the filter barrier. The transmission probability *T* through the filter can be increased by increasing the energy of the ballistic electrons, using resonant tunneling energies with unity transmission or by using a nonrectangular filter profile.^33^ In particular, engineering a sophisticated multilayer structure^34^ in analogy to the antireflection coatings in the optical domain is an appealing approach to obtain near-unity transmission over a wider energy window.

**Figure 5:**
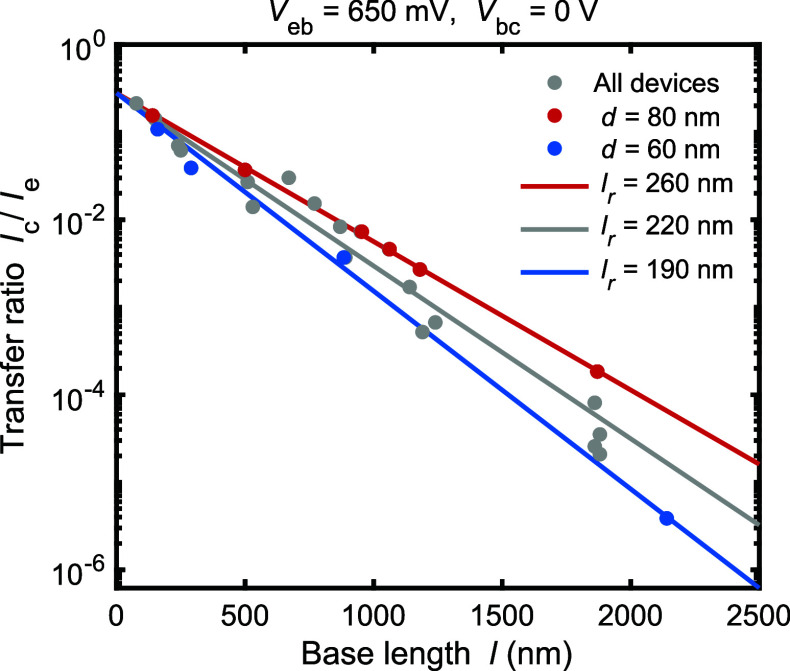
Base length *l* dependence of transfer ratio *I*_c_/*I*_e_. Dots are experimental data from several devices, and the solid lines are fits to exponential dependence *I*_c_/*I*_e_ = *T* exp(−*l*/*l*_r_). The red and blue data show the results with nanowires sorted to two diameters: *d* = 60–65 nm and 76–82 nm, respectively.

With the red and blue points in Figure 5, we sorted the devices into two categories with different diameters *d*. We observe that the majority of the scattering of the data is explained by diameter dependence, with smaller diameter *d* = 60 nm leading to shorter mean-free path of *l*_r_ = 190 nm compared to larger diameter *d* = 80 nm with *l*_r_ = 260 nm. This finding suggests that surface scattering contributes at least partially to the relaxation. At small *l*, both curves meet, indicating a diameter-independent transmission probability *T*. Comparing the observed mean-free path to theoretical predictions, we used the conventional Fröhlich coupling within the two-band Kane model.^35,36^ This calculation results in a phonon relaxation rate of *l*_r_ = 230 nm, in excellent agreement with the experiments; see methods for details. The nonparabolicity of the conduction band is important for the relaxation length: neglecting it results in five times larger *l*_r_, which is not consistent with the experiments.

In conclusion, we demonstrated transport in the ballistic limit with three-terminal hot carrier nanowire devices. The structures were made with 1D epitaxy, enabling flexible bandgap engineering with smooth and sharp interfaces without doping. We determined the mean-free path and barrier transmission probability directly from the experiments at room temperature. For the shortest devices, most of the electrons (70%) are ballistic. We also found the mean-free path to depend on the nanowire diameter. These findings are consistent with theoretical estimates.

## Supporting Information Available

Additional information about the nanowire heterostructure growth, device processing, energy relaxation calculations with the Kane model, and transmission probability estimation (PDF)
