# Supplementary material for: Hot Carrier Nanowire Transistors at the Ballistic Limit
Source: Nano Lett. 2024 Jun 24;24(26):7948–52. doi: 10.1021/acs.nanolett.4c01197 (PMC11229068; doi:10.1021/acs.nanolett.4c01197)
Supplement: Supplementary file 1 — nl4c01197_si_001.pdf [file nl4c01197_si_001.pdf]

Supplemental material for the article "Hot Carrier Nanowire Transistors at the Ballistic Limit"

Mukesh Kumar,[1] Ali Nowzari,[1] Axel R. Persson,[2] Sören Jeppesen,[1] Andreas Wacker,[3] Gerald Bastard,[4] Reine L. Wallenberg,[2] Federico Capasso,[5] Ville F. Maisi,[1] and Lars Samuelson[1, 6]

[1]*NanoLund and Division of Solid State Physics, Lund University, Box 118, 22100 Lund, Sweden*
[2]*NanoLund and Centre for Analysis and Synthesis, Lund University, Box 117, 22100, Lund, Sweden*
[3]*NanoLund and Division of Mathematical Physics, Lund University, Box 118, 22100 Lund, Sweden*
[4]*Physics Department ENS-PSL, 24 rue Lhomond F75005, France*
[5]*John A. Paulson School of Engineering and Applied Sciences, Harvard University, 9 Oxford Street McKay Labs Room 125, Cambridge, MA 02138, United States*
[6]*Institute of Nanoscience and Applications, Southern University of Science and Technology, 1088 Xueyuan Avenue, Shenzhen 518055, China*

NANOWIRE HETEROSTRUCTURE GROWTH

Various growth stages in chemical beam epitaxy (CBE) system were systematically followed to grow the InAs/InP based nanowire heterostructures using Au aerosol particles (diameter ∼ 60 nm) deposited on InAs(111)B substrates. The precursors used were trimethylindium (TMIn), precracked tertiarybutylarsine (TBAs) and precracked tertiarybutylphosphine (TBP) for In, As and P, respectively. The first growth step was the removal of surface oxides from substrate which was carried out through annealing of the substrate at 540 °C under As pressure. Then the substrate temperature was lowered and stabilized to 420 °C. Pressures for different sources in the lines were controlled and measured prior to their entrance in growth chamber. Onset of growth is defined through flowing In into the chamber with fixed flow of TBAs. First, a InAs stem was grown using 0.1 mbar TMIn and 1.5 mbar TBAs. Subsequently, substrate temperature was further lowered and stabilized at 400 °C. Next, the operational segments of the nanowire heterostructure were grown at this stabilized temperature. The segments were grown by controlling the length with growth time in the following order: InAs emitter, $InAs_{1-x}P_x$, based graded barrier, InAs base, InP based filter barrier and InAs collector. The composition between different segments was set by changing and switching the partial pressures of the precursors. The compositional grading was grown using a constant TMIn pressure of 0.1 mbar, constant TBP pressure of 1 mbar and varying the TBAs pressure from 1.5 mbar to the withdrawal from the chamber. To limit tunnelling through the sharp triangular top of the injector barrier the highest phosphorous content based ternary was extended at fixed composition by about a few 10's of nm before a sharp termination of the barrier. After growing the subsequent InAs segment defining the flight length l for the electrons, the rectangular energy filter barrier was grown with InAsP heterostructure segment in a nanowire. Figure S1 shows the measured elemental composition profile of the nanowire of Fig. 1 of the main manuscript. The growth conditions required careful tuning to provide the right ternary composition, sharp hetero-interfaces, correct dimensions, and to maintain the entire heterostructure in a straight axial morphology. Therefore, a number of growth runs were performed to obtain devices with different base length l.

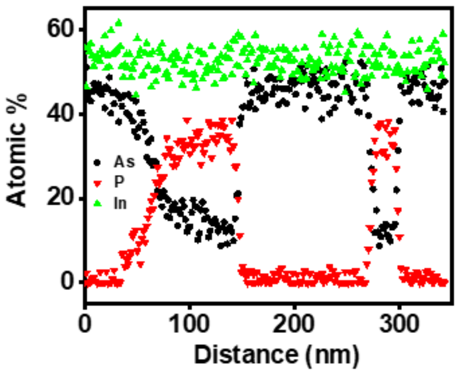

FIG. S1. Elemental composition profile measured with Energy Dispersive X-Ray Spectroscopy (EDS) along the nanowire.

NANOWIRE DEVICE PROCESSING

After the growth, the three terminals were made to form the hot electron nanowire devices. The processing steps are as follows: The grown nanowires were transferred to a cleaned Si substrate capped with a 100 nm thick thermal oxide. The substrate was additionally pre-processed to have the lithographically defined alignment marks and metal pads for electrical contacts. Af-

ter nanowire transfer, their location with respect to the alignement marks was determined and an electron beam lithography was performed to contact the wires. Just before metallization, ammonium polysulfide ($(NH_4)_2S_x$) solution based process was employed to remove the native oxide from the exposed InAs leads as well as to passivate the surface to avoid reoxidation [1]. Ohmic metal contacts (25 nm Ni and 125 nm Au) to the InAs leads were deposited by thermal evaporation followed by lift-off.

ENERGY RELAXATION WITH THE KANE MODEL

Energy dispersion: To consider the energy relaxation in the base electrode, we use the so-called Kane model [2, 3] implying the dispersion

$$E(k) = E_c + \frac{-\Delta}{2} + \frac{\Delta}{2}\sqrt{1 + 2\frac{\hbar^2 k^2}{m^*\Delta}} = E_c + \frac{\hbar^2 k^2}{2\,m(E)}, \quad (1)$$

where E_c is the conduction band edge, $m(E) = m^*(1 + (E - E_c)/\Delta))$ the energy dependent effective mass, m^* the effective mass at the conduction band edge and Δ the energy gap. As we consider energies far from the conduction band edge, we take non-parabolicity into account in Eq. (1). This provides the energy dependence of the quasi-momentum and velocity as

$$\begin{cases} k(E) &= \frac{1}{\hbar}\sqrt{2m^*(E - E_c)\left(1 + \frac{E-E_c}{\Delta}\right)} \\ v(E) &= \frac{\hbar k(E)}{m^*}\left(1 + 2\frac{E-E_c}{\Delta}\right)^{-1}. \end{cases} \quad (2)$$

We use $E_c = 0$, $\Delta = 0.354$ eV and $m^* = 0.023\,m_e$ for InAs as well as $E_c = \Phi_B = 0.5$ eV, $\Delta = 1.344$ eV and $m^* = 0.08\,m_e$ for InP barrier. For the kinetic energy of $E = 0.5$ eV in the InAs base contact, Eq. (2) yields a velocity of $v(E) = 1.12 \times 10^6$ m/s. For comparison, using the standard parabolic bandstructure in the conduction band, we obtain $v(E) = \sqrt{2E/m^*} = 2.77 \times 10^6$ m/s, which demonstrates the huge impact of non-parabolicity.

Phonon scattering: The scattering rate for the spontaneous emission of polar optical phonons via the Fröhlich interaction is

$$W^{\text{polar LO}}_{\mathbf{k}\to\mathbf{k}'} = \frac{2\pi}{\hbar}\frac{\hbar^2 A_{\text{Fr}}}{V}\frac{\delta\left(E(\mathbf{k}') - E(\mathbf{k}) + \hbar\omega_{LO}\right)}{|\mathbf{k} - \mathbf{k}'|^2} \quad (3)$$

with the material constant

$$A_{\text{Fr}} = \frac{\omega_{LO}e^2}{2\epsilon_0\hbar}\left(\frac{1}{\epsilon(\infty)} - \frac{1}{\epsilon_r}\right) = 9.58 \times 10^{18}\frac{\text{m}}{\text{s}^2}.$$

Here we use the optical phonon energy $\hbar\omega_{LO} = 30$ meV, the static dielectric constant $\epsilon_r = 15.15$, as well as its high frequency value $\epsilon(\infty) = 12.3$ which are common values for bulk InAs. For an isotropic band structure $E(\mathbf{k}) = E(k)$ with the Kane model addressed above, we obtain the total spontaneous LO phonon emission rate for an electron in the initial state $\mathbf{k}$ as

$$\begin{aligned} \frac{1}{\tau_{\text{LO emis.}}} &= \frac{V}{(2\pi)^3}\int \mathrm{d}^3k'\, W^{\text{polar LO}}_{\mathbf{k}\to\mathbf{k}'} = \\ &\frac{A_{\text{Fr}}m^*}{2\pi\hbar}\,\frac{1+2(E_0-\hbar\omega_{LO})/\Delta}{k}\log\left|\frac{k+k_f}{k-k_f}\right|\Theta(E_0 - \hbar\omega_{LO}), \end{aligned} \quad (4)$$

where the momenta k, k_f depend on the initial energy E_0 as

$$\begin{cases} \hbar k &= \sqrt{2m^*E_0 + 2m^*\frac{E_0^2}{\Delta}} \\ \hbar k_f &= \sqrt{2m^*(E_0 - \hbar\omega_{LO}) + 2m^*\frac{(E_0-\hbar\omega_{LO})^2}{\Delta}}. \end{cases} \quad (5)$$

This provides the scattering time $\tau_{\text{LO emis.}} = 207$ fs for the initial energy $E_0 = 0.5$ eV at room temperature. Together with the velocity of 1.12×10^6 m/s addressed above, we obtain a relaxation length $l_r = 230$ nm. Here we disregard the stimulated processes as the energy balance for stimulated emission and absorption approximately compensate each other. Given the fact that the scattering mostly results only in small changes in the Bloch vector, see Eq. (3), the velocity is hardly changed and the net energy loss by spontaneous emission dominates the odds of the injected electron to overcome the barrier. We note, that the use of a parabolic band provides more than twice the scattering time, due to the reduced density of states. In combination with the increased velocity, this provides relaxation lengths which are about five times larger and not consistent with the experimental findings.

Impurity scattering: We use the common screened impurity potential

$$V(\mathbf{r}) = \frac{e^2 \mathrm{e}^{-\lambda r}}{4\pi\varepsilon_r\epsilon_0 r} = \frac{1}{V}\sum_{\mathbf{q}}\frac{e^2}{\varepsilon_r\epsilon_0(q^2 + \lambda^2)}\mathrm{e}^{\mathrm{i}\mathbf{q}\cdot\mathbf{r}} \quad (6)$$

with the Debye screening length $\lambda^2 = ne^2/\varepsilon_r\epsilon_0 k_B T$, where n is the electron density and we apply the temperature $T = 300$ K. Fermi's golden rule provides for N_i independent impurities

$$\begin{aligned} \frac{1}{\tau_{\text{imp}}} &= N_i\frac{2\pi}{\hbar}\frac{V}{(2\pi)^3}\int \mathrm{d}^3k' \\ &\left|\frac{e^2}{V\varepsilon_r\epsilon_0(|\mathbf{k}-\mathbf{k}'|^2+\lambda^2)}\right|^2\delta\big(E(\mathbf{k}) - E(\mathbf{k}')\big) \\ &= \frac{N_i}{V}\frac{e^4}{4\pi\hbar^2\varepsilon_r^2\epsilon_0^2}\frac{1}{v(k)}\left(\frac{1}{\lambda^2} - \frac{1}{4k^2+\lambda^2}\right). \end{aligned} \quad (7)$$

Note that $\frac{N_i}{V\lambda^2}$ does not depend on the doping in case of charge neutrality $n = N_i/V$, which is assumed here. For k corresponing to the initial energy of 0.5 eV, we obtain with the Kane model $\tau_{\text{imp}} = 198$ fs for a doping density of $10^{17}/\text{cm}^3$. This is very similar to the spontaneous phonon emission time calculated above. However most of the scattering is forward and the common momentum relaxation time 11.5 ps is significantly longer, so that impurity scattering should not limit the transmission over

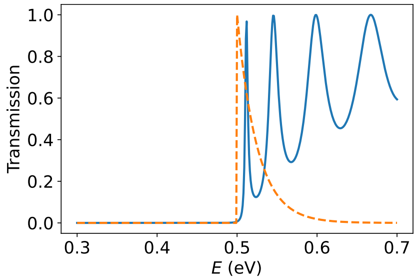

FIG. S2. Transmission (full line) through the InP barrier with a thickness of $a = 20$ nm using the Kane model together with the thermal distribution (dashed line) at room temperature, which is used for averaging.

the barrier. This result is obtained by inserting the factor $(1 - \cos[\angle(\mathbf{k}, \mathbf{k}')])$ into the integral in Eq. (7).

Electron-electron scattering matrix elements are structurally similar to the impurity scattering, but due to exchange effects one expects a reduction of scattering rate by a factor of four [4, 5]. Thus we neglect these here. An open issue are Auger processes, which we did not consider.

The effect of crystal structure: Above we applied the parameter values for bulk InAs, which are well established. A major issue is the fact, that the experimental nanowires are of wurtzite type. Band structure calculations in Ref. 6 provide a linear energy-k relation with $v_D \approx 0.9 \times 10^6$ m/s in the range of interest, which is quite comparable to the zincblende case: Reference 7 reports $\omega_{LO} = 2\pi c \cdot 239/\text{cm}$, which agrees well with the zincblende value of ω_{LO}. Furthermore $\omega_{TO} = 2\pi c \cdot 214/\text{cm}$, provides a ratio $\omega_{LO}/\omega_{TO} = 1.12$, which agrees well with the Lyddane-Sachs-Teller relation $\sqrt{\varepsilon_r/\varepsilon(\infty)} = 1.11$ for zincblende. A slightly smaller value $\omega_{LO}/\omega_{TO} = 236/219 = 1.08$ is reported in Ref. 8. In summary, the scattering rate should not differ significantly between wurtzite and zincblende material.

Finally, it is worth to mention that the splitting between the first and second conduction band in wurtzite InAs nanowires was recently measured to equal to 590 meV [9], which might provide complications not considered here.

TRANSMISSION PROBABILITY

The transmission through the barrier of thickness d can be evaluated in a standard way [10], resulting in the transmission probability

$$T(E) = \left[1 + \left|\frac{m_w k_b}{m_b k_w} - \frac{m_b k_w}{m_w k_b}\right|^2 |\sin(k_b a)|^2\right]^{-1}, \qquad (8)$$

where the $m_{w/b}$ and $k_{w/b}$ are the functions $m(E)$ and $k(E)$ given in Eqs. (1) and (2) with InAs and InP parameters respectively. Note, that the expression holds both for $E > \Phi_B$ and for $E < \Phi_B$ with k_b being imaginary. The result is shown in Fig. S2 with thermal Bolzmann-distribution $\propto \exp(-E/k_B T)$ for $E > \Phi_B$.

REFERENCES

[1] D. B. Suyatin, C. Thelander, M. T. Björk, I. Maximov, and L. Samuelson, Sulfur passivation for ohmic contact formation to InAs nanowires, Nanotechnology **18**, 105307 (2007).

[2] S. R. White and L. J. Sham, Electronic properties of flat-band semiconductor heterostructures, Phys. Rev. Lett. **47**, 879 (1981).

[3] G. Bastard, Superlattice band structure in the envelope-function approximation, Phys. Rev. B **24**, 5693 (1981).

[4] M. Dür, S. M. Goodnick, and P. Lugli, Monte Carlo simulation of intersubband relaxation in wide, uniformly doped GaAs/Al$_x$Ga$_{1-x}$As quantum wells, Phys. Rev. B **54**, 17794 (1996).

[5] H. Callebaut, S. Kumar, B. S. Williams, Q. Hu, and J. L. Reno, Importance of electron-impurity scattering for electron transport in terahertz quantum-cascade lasers, Appl. Phys. Lett. **84**, 645 (2004).

[6] P. E. Faria Junior, T. Campos, C. M. O. Bastos, M. Gmitra, J. Fabian, and G. M. Sipahi, Realistic multiband $k \cdot p$ approach from ab initio and spin-orbit coupling effects of InAs and InP in wurtzite phase, Phys. Rev. B **93**, 235204 (2016).

[7] M. Möller, M. M. de Lima, A. Cantarero, L. C. O. Dacal, J. R. Madureira, F. Iikawa, T. Chiaramonte, and M. A. Cotta, Polarized and resonant raman spectroscopy on single InAs nanowires, Phys. Rev. B **84**, 085318 (2011).

[8] S. D. Dabhi and P. K. Jha, Phonon dispersion and raman spectra of wurtzite InAs under pressure, J. Phys. Chem. Solids **83**, 70 (2015).

[9] S. Pournia, S. Linser, G. Jnawali, H. E. Jackson, L. M. Smith, A. Ameruddin, P. Caroff, J. Wong-Leung, H. H. Tan, C. Jagadish, and H. J. Joyce, Exploring the band structure of wurtzite InAs nanowires using photocurrent spectroscopy, Nano Research **13**, 1586 (2020).

[10] J. H. Davies, The physics of low-dimensional semiconductors (Cambridge University Press, 2006).
